# Identification of Ocular Autoantigens Associated With Juvenile Idiopathic Arthritis-Associated Uveitis

**DOI:** 10.3389/fimmu.2019.01793

**Published:** 2019-08-06

**Authors:** Martin Busch, Kira Leona Wefelmeyer, Karoline Walscheid, Kai Rothaus, Dirk Bauer, Cornelia A. Deeg, Roxane L. Degroote, Doreen Ackermann, Simone König, Solon Thanos, Maren Kasper, Arnd Heiligenhaus

**Affiliations:** ^1^Ophtha-Lab, Department of Ophthalmology at St. Franziskus Hospital, Münster, Germany; ^2^Department of Ophthalmology at St. Franziskus Hospital, Münster, Germany; ^3^Chair of Animal Physiology, Department of Veterinary Sciences, Ludwig-Maximilians-University of Munich, Munich, Germany; ^4^IZKF Core Unit Proteomics, University of Münster, Münster, Germany; ^5^Institute of Experimental Ophthalmology, University of Münster, Münster, Germany; ^6^University of Duisburg-Essen, Essen, Germany

**Keywords:** autoantibodies, autoantigens, juvenile idiopathic arthritis, uveitis, proteomics, autoimmunity

## Abstract

The purpose of the current study was to analyze the binding patterns of serum autoantibodies from juvenile idiopathic arthritis (JIA) and JIA-associated uveitis (JIAU) patients to proteomes from different ocular tissues and to identify potential ocular autoantigens in JIAU. Proteomes from porcine iris, ciliary body, or retina tissue were isolated, separated using 2D-gel electrophoresis, and transferred to a blotting membrane. The binding pattern of serum antibodies from JIA or JIAU patients or healthy controls to ocular proteins was visualized by using anti-human IgG secondary antibodies and chemiluminescence reaction. Selected protein spots were excised from silver-stained 2D gels and subjected to mass spectrometry. Serum antibodies binding to ocular proteins were detected in all patient groups and healthy controls. Irrespective of the patient groups, serum antibodies bound to 49 different protein spots of the retina proteome, to 53 of the ciliary body proteome, and to 44 of the iris proteome. The relative binding frequency of sera to these iris protein spots was significantly higher in JIAU than in JIA patients or healthy controls. Particularly in JIAU patients, cluster analyses indicated a broad range of serum antibodies directed against ocular antigens, mostly in the iris proteome. Iris proteins frequently bound by serum antibodies in all groups were identified as tubulin beta chain, vimentin, ATP synthase subunit beta, actin, and L-lactate dehydrogenase B chain. Iris proteins exclusively bound by JIAU serum antibodies were heat shock cognate 71 kDa protein and keratin. Although serum autoantibody binding to ocular antigens was not disease-specific, a significant diversity of autoantibodies against a broad range of antigens, particularly from the iris tissue, was detected in JIAU patients. As the iris is a major site of inflammation in JIAU, the present data give further evidence that autoantibodies may be involved in JIAU immunopathology.

## Introduction

Juvenile idiopathic arthritis (JIA) defines a heterogeneous group of chronic inflammatory rheumatic diseases with onset prior to an age of 16 years (1) and it is an important cause of articular damage (2). According to the International League of Associations for Rheumatology (ILAR) criteria, JIA is classified into seven subtypes, comprising systemic arthritis, oligoarthritis (persistent and extended), rheumatoid factor (RF)-negative and -positive polyarthritis, psoriatic arthritis, enthesitis-related arthritis, and undifferentiated arthritis (1).

Uveitis in children and adolescents is frequently related to JIA (3–5), and, vice versa, represents a common extra-articular manifestation of disease (5, 6). JIA-associated uveitis (JIAU) occurs in 9 to 12% of children with JIA and is observed particularly frequently in the oligoarthritis subtype (7, 8).

JIAU typically presents as anterior uveitis encompassing inflammation and inflammatory cell infiltration predominantly in the iris and ciliary body of the eye according to the Standardization of Uveitis Nomenclature (SUN) classification (9); it is chronic and bilateral, and the onset of flare is insidious (3, 4, 8). JIAU patients are at high risk for developing sight-threatening complications, which requires frequent ophthalmologic examination and the use of corticosteroids and disease-modifying anti-rheumatic drugs (DMARDs) (10, 11).

Although inflammatory autoimmune reactions involving adaptive and innate immune cells interacting in a versatile network are suspected (12), the precise mechanisms underlying JIAU pathogenesis are not well-understood. There is increasing evidence indicating that B and plasma cells, representing the predominant cellular infiltrate, play a crucial role in the disease (13–16). This hypothesis is supported by the observation that 80% of JIAU patients are positive for serum anti-nuclear antibodies (ANA), which represent an important risk factor for uveitis occurrence in JIA patients (6, 8). However, the pathogenic contribution of ANA or autoantibodies to JIAU is still largely undefined.

In the present study, by employing 2D-polyacrylamide gel electrophoresis (2D PAGE), western blotting, and mass spectrometry (MS), we analyzed binding patterns of serum autoantibodies to proteomes isolated from porcine ocular tissues as a model to study the autoimmune target structures in JIAU.

## Materials and Methods

### Patients and Sample Collection

Peripheral blood serum samples were collected at the Department of Ophthalmology at St. Franziskus Hospital Muenster (Germany) between 2008 and 2015. JIA and JIAU patients included in the study conformed to JIA criteria according to ILAR (1) and rank among the oligoarthritis subtype, the one most commonly causative for uveitis (8). JIAU patients suffered from chronic anterior uveitis classified according to the SUN criteria (9). Patients included in the JIA control group also had undergone regular ophthalmologic examination in accordance with the current German screening recommendations (8), and had at no time shown any signs of ocular involvement. Using a standardized ophthalmic database, the age at time of sampling, the age at JIA onset, gender, ANA, RF, and HLA-B27 status as well as any anti-inflammatory treatments were recorded. Furthermore, in the JIAU group, best-corrected visual acuity (BCVA), uveitis activity according to the anterior chamber (AC) cell grade (9), and secondary eye complications (e.g., cataract, glaucoma, and macular edema) were documented. Children without any inflammatory joint or eye disease subjected to elective otorhinolaryngologic surgery served as further controls.

Venous blood was taken from the children and centrifuged at 699 × *g* for 10 min. Serum was taken and stored at −80°C until use.

Sample collection and study procedure complied with the principles of the Declaration of Helsinki and the study was approved by the local ethics committee (approval number: 2009-156-f-S). Written informed consent from those patients aged 14 years or older and, additionally, patients' parents (in all patients younger than 18 years of age) was obtained before study entry.

### Study Design

Proteomes from iris, ciliary body, or retina tissues from porcine eyes were isolated and separated using 2D PAGE. Afterwards, the proteins were transferred onto a blotting membrane, which was then incubated with serum either from JIAU patients, JIA patients, or healthy controls. Binding of serum antibodies to the proteins on the membrane was detected and visualized by using anti-human IgG secondary antibodies and chemiluminescence. Serum antibody binding patterns were analyzed, quantified, and compared between the patient groups. To identify potential autoantigens, serum antibody-bound protein spots of interest were traced on silver-stained master gels, excised, and further analyzed by MS.

### Protein Isolation

Iris, ciliary body, or neuroretina were isolated from eyes of newborn pigs (2 days postnatally) immediately after slaughtering. Before protein isolation, each of the tissues from several eyes was pooled. The tissue specimens were then homogenized in urea lysis buffer (9 M urea, 2 M thiourea, 65 mM CHAPS, and 65 mM DTT) supplemented with 1 mM PMSF (SERVA, Heidelberg, Germany) and protease inhibitor cocktail (Complete, EDTA-free protease inhibitor cocktail, Roche Diagnostics, Mannheim, Germany) using ReadyPrep Mini Grinders according to the manufacturer's instructions (Bio-Rad, Munich, Germany). After centrifuging at 17,000 × *g* for 30 min, the supernatants were collected and stored at −80°C until isoelectric focusing. The protein concentration was determined by using a commercially available Bradford protein assay (Bio-Rad).

## 2D Page

For first-dimension separation of proteins by isoelectric focusing (IEF), immobiline dry strips with an immobilized non-linear pH gradient were used (IPG strips, pH3-11 NL, 11 cm, GE Healthcare, Freiburg, Germany). IPG strips were rehydrated overnight in rehydration buffer (7 M urea, 2 M thiourea, 4% CHAPS (w/v), 1% Pharmalyte pH3-10 (v/v), 0.2% DTT (w/v), and 0.004% bromophenolblue (w/v) containing 7.5 μg protein/sample. An IEF system (Ettan IPGphor II, GE Healthcare) with a 7.5 h IEF program was used as follows: step 1 (150 V for 1 h; voltage mode: step); step 2 (300 V for 1 h; voltage mode: step); step 3 (1,000 V for 1 h; voltage mode: gradient); step 4 (6,000 V for 2 h; voltage mode: gradient); step 5 (6,000 V for 2.5 h; voltage mode: step); and step 6 (300 V, step and hold). For second-dimension separation of proteins using sodium dodecylsulfate (SDS)-PAGE, the IPG strips were equilibrated for 10 min in SDS equilibration buffer supplemented with 1% DTT (w/v), followed by incubation in equilibration buffer supplemented with 4.8% iodoacetamide (w/v) for another 10 min. Equilibrated IPG strips were placed on top of a 8–16% SDS-polyacrylamide gradient gel (7 ×8 cm, SERVA, Heidelberg, Germany) positioned in a vertical electrophoresis system (SE260 Mighty Small II Deluxe, Hoefer, Holliston, USA). SDS-PAGE was run at 60 V, 80 mA, and 1 W for 30 min followed by 175 V, 120 mA, and 10 W for 2 h using the Laemmli electrophoresis buffer system (17). The 2D gels were either used for immunoblotting or as master gels for identifying proteins by MS. Master gels were stained using an MS-compatible silver staining kit (SERVA Silver Staining Kit SDS-PAGE, SERVA).

### Immunoblotting

The proteins isolated from iris, ciliary body, or retina tissues and separated by 2D PAGE were transferred onto a PVDF membrane (GE Healthcare) at 256 V, 318 mA, and 50 W for 40 min by using a semi-dry blotting technique. Afterwards, the membrane was blocked with 1% (w/v) polyvinylpyrrolidone (PV40, Sigma-Aldrich, Taufkirchen, Germany) in PBS supplemented with 1% (v/v) rabbit serum (DakoCytomation, Hamburg, Germany) for 1 h. The membrane was then incubated with patient serum diluted 1:1,000 in PBS and 0.1% Tween (PBST) at 4°C overnight. An individual membrane was used for each serum sample (JIAU, *n* = 23; JIA, *n* = 14; healthy control, *n* = 10). Specific binding of serum antibodies was detected by incubating the membrane with secondary horseradish peroxidase-conjugated rabbit anti-human IgG antibody diluted 1:30,000 in PBST with 1% porcine serum (DakoCytomation, Hamburg, Germany) for 1 h followed by incubation with chemiluminescent substrate (SuperSignal West Pico Chemiluminescent Substrate; Thermo Scientific, Rockford, IL, USA). Each step was followed by several washes in PBST. The resulting chemiluminescent reaction was visualized and documented by using the ChemiDoc^TM^ XRS+ imaging system (Bio-Rad). Afterwards, the membrane was incubated with colloid gold staining solution (Sigma-Aldrich).

### Data Acquisition and Statistics

**Figure 1:**
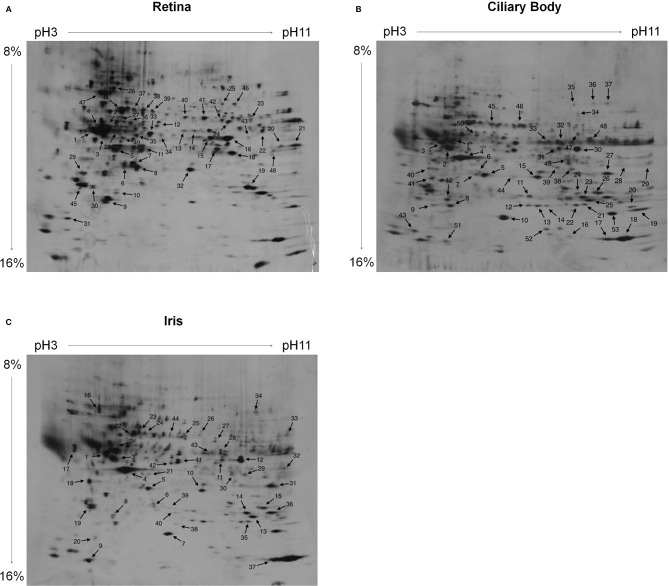
Silver-stained 2D PAGE master gels. The proteomes of **(A)** retina, **(B)** ciliary body, and **(C)** iris tissue were separated. For IEF first-dimension separation, IPG strips ranging from pH3-11 (non-linear) were used. For SDS-PAGE, 8–16% polyacrylamide gradient gels were used. Protein spots that reacted with patient serum antibodies are labeled on the master gels. Irrespective of the patient group, the sera collectively reacted with 49 protein spots of the retina proteome **(A)**, with 53 protein spots of the ciliary body proteome **(B)**, and with 44 protein spots of the iris proteome **(C)**.

Serum antibody-bound protein spots visualized on immunoblots were traced on gold-stained blots that reflected the entire proteome by generating an overlay of immunoblot and gold staining from the same blot. Thus, the gold staining assured equivalent protein separation and the transfer to the blotting membranes, and served to assign the detected spots in the immunoblots to corresponding spots on a silver-stained master gel. Irrespective of the serum patient group, each protein spot that was detected by immunoblotting and traced in the proteome was marked on a silver-stained master gel (Figure 1). Thereby, the applied procedure was modified according to previous studies (18, 19).

For each marked protein spot, the proportion of sera of each patient group binding the respective protein was determined and defined as relative binding frequency. Additionally, the number of protein spots bound to by an individual patient's serum was calculated.

The IBM SPSS Statistics version 23.0.0.0 (IBM Corporation, Armonk, NY, USA), the MedCalc v10.0.1.0 (MedCalc Software bvba, Ostend, Belgium), and the statistic software R (Version 3.2.5, distributed by CRAN) were used to analyze the data. All data, including demographic patient data, were tested for normal distribution and homogeneity of variance and analyzed by conducting parametric or non-parametric tests, as appropriate. Binding of sera to single protein spots was analyzed with the Chi^2^ test.

### Mass Spectrometry Analysis

To identify antigenic proteins isolated from the ocular tissues and bound by serum antibodies, selected protein spots were excised from a silver-stained 2D master gel and subjected to mass spectrometry (MS).

Proteins were reduced, alkylated, and tryptically digested in the gel. Peptides were extracted, dried, and redissolved in 5 μl 0.1% formic acid containing 5% acetonitrile. High-definition MS was performed using a Synapt G2 Si ion mobility mass spectrometer coupled to M-Class UPLC (Waters Corp.) with a 30-min gradient (solvent system 100% water vs. 100% acetonitrile, both containing 0.1% formic acid; trap column V/M Symmetry C18 100 Å 5 μm, 180 μm ×20 mm; reversed phase column HSS T3 1.8 μm, 75 μm ×200 mm; and 4.5 μl injection volume). Data were analyzed with PLGS software (Waters Corp.) using the human and pig UniProt databases for protein identification.

## Results

### Demographic and Clinical Characteristics of the Patients

JIA patients without uveitis (JIA; *n* = 14) and patients with JIA-associated uveitis (JIAU; *n* = 23) included in the study had similar characteristics: the majority of patients was ANA-positive, RF-negative, and suffered from the JIA oligoarthritis (persistent/extended) subtype. At the time of sampling, more JIAU than JIA patients had received systemic anti-inflammatory therapy (*p* = 0.03). JIAU patients included in the study had chronic bilateral anterior uveitis, which was active [anterior chamber cells ≥1+ (9)] in 26% of patients at the time of sampling. In 60% of the JIAU patients, uveitis was associated with complications, among which synechiae, cataract, and glaucoma were most frequent. At the time of sampling, 43.5% of the JIAU patients already had eye surgery previously. A further group of healthy controls (*n* = 10) without any inflammatory joint or eye disease was included in the study. The mean age in the healthy control group was significantly lower than in the JIA and JIAU group. No difference was observed between the groups for sex ratio, which was slightly shifted toward female gender. Clinical data of patient groups and controls are summarized in Table 1.

**Table 1:** Patient data.

	**Healthy controls (*n* = 10)**	**JIA (*n* = 14)**	**JIAU (*n* = 23)**	***P*-value**
Age at sampling in years (mean ± SD)	8.4 ± 3.0	13.4 ± 3.0	11.6 ± 4.7	0.009^a^
JIA disease duration in years (mean ± SD)	n.a.	3.5 ± 2.8^b^	8.3 ± 5.1	0.004^c^
Uveitis duration in years (mean ± SD)	n.a.	n.a.	7.0 ± 4.1	
Male sex [*n* (%)]	4 (40)	6 (42.9)	6 (26.1)	0.35^d^
ANA positivity [*n* (%)]	n.a.	14 (100)	21 (91.3)	0.257^d^
RF positivity [*n* (%)]	n.a.	0 (0)	0 (0)	
Topical corticosteroids	n.a.	n.a.	11 (47.8)	
Any systemic corticosteroid and/or DMARD treatment	n.a.	10 (71.4)	23 (100)	0.03^d^
Systemic corticosteroids	n.a.	2 (14.3)	5 (21.7)	0.898^d^
Conventional synthetic DMARDs^e^	n.a.	10 (71.4)	23 (100)	0.03^d^
Biologic DMARDs^f^	n.a.	0 (0)	7 (30.4)	0.063^d^
Chronic anterior uveitis, bilateral [*n* (%)]	n.a.	n.a.	23 (100)	
Uveitis activity: anterior chamber cells ≥ 1 [*n* (%)]	n.a.	n.a.	6 (26)	
Ocular complications (total) [*n* (%)]	n.a.	n.a.	14 (60.9)	
Cataract [*n* (%)]	n.a.	n.a.	10 (43.5)	
Glaucoma or ocular hypertension [*n* (%)]	n.a.	n.a.	8 (34.8)	
Synechiae [*n* (%)]	n.a.	n.a.	10 (43.5)	
Vitreous haze [*n* (%)]	n.a.	n.a.	2 (8.7)	
Band keratopathy [*n* (%)]	n.a.	n.a.	5 (21.7)	
Papillary edema [*n* (%)]	n.a.	n.a.	5 (21.7)	
Previous eye surgery [*n* (%)]	n.a.	n.a.	10 (43.5)	
BCVA left eye (logMAR)	n.a.	n.a.	0.35	
BCVA right eye (logMAR)	n.a.	n.a.	0.22	
Oligoarthritis, persistent [*n* (%)]	n.a.	14 (100)	20 (87)	0.159^d^
Oligoarthritis, extended [*n* (%)]	n.a.	0 (0)	3 (13)	0.43^d^

a *Kruskal-Wallis test (post-hoc analysis: healthy control different from JIA (p <0.05) and JIAU (p <0.05))*.

b *recorded for n = 12 JIA patients*.

c *Mann-Whitney test*.

d *Chi^2^ test*.

e *Azathioprine (JIA, n = 0; JIAU, n = 2), CsA (JIA, n = 0; JIAU, n = 3), Hydroxychloroquine (JIA, n = 3; JIAU, n = 0), Methotrexate (JIA, n = 7; JIAU, n = 21)*.

f *Abatacept (n = 1), Adalimumab (n = 6)*.

*ANA, antinuclear antibodies; BCVA, best-corrected visual acuity; DMARDs, disease-modifying anti-rheumatic drugs; JIA, juvenile idiopathic arthritis; JIAU, juvenile idiopathic arthritis-associated uveitis; logMAR, Logarithm of the Minimal Angle of Resolution; n.a., not applicable; RF, rheumatoid factor; SD, standard deviation*.

### Serum Antibody-Binding Patterns to Proteins Isolated From Ocular Tissues

By using immunoblotting, binding of serum antibodies to the proteome of ocular tissues (iris, ciliary body, and retina) was analyzed. Protein spots bound by serum antibodies of JIA, JIAU, or control sera were collectively marked on a 2D master gel (Figure 1).

In all three proteomes, the mean number of protein spots bound to by individual serum samples did not differ between the patient groups and did not correlate to the incidence of ocular complications in JIAU patients (data not shown).

**Figure 2:**
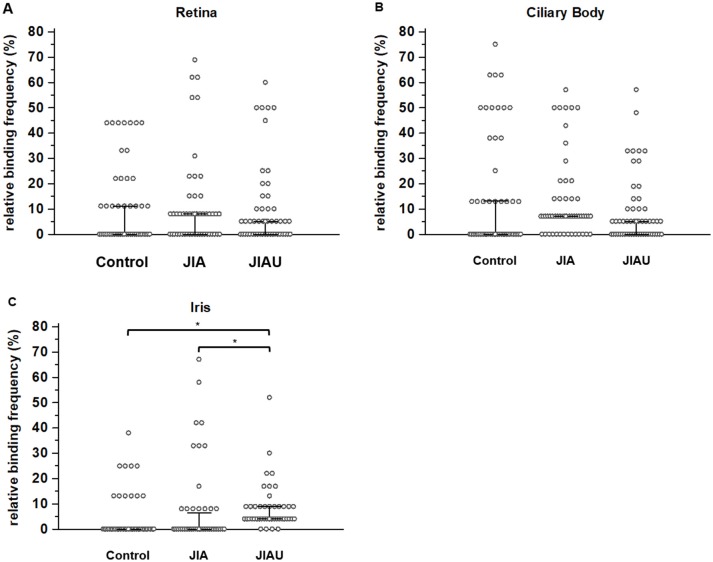
The relative binding frequency is defined as the proportion of sera that reacts with the respective protein spot. The relative binding frequency is shown for each of the protein spots labeled on the **(A)** retina proteome (*n* = 49), **(B)** ciliary body proteome (*n* = 53), and **(C)** iris proteome (*n* = 44) and compared between the different patient groups. Error bars indicate medians and corresponding 95% confidence intervals of medians. *p < 0.05 (Kruskal-Wallis test with *Post-hoc* analysis).

With regard to the spots marked on the retina proteome, no differences in the relative binding frequency of sera were determined between the patient groups. The same was true for the binding of sera to the ciliary body proteome. However, regarding the protein spots marked on the iris proteome, the relative binding frequency of JIAU sera was significantly higher than for JIA and healthy control sera (Figure 2).

We also analyzed the relative binding frequency within the patient groups to the different proteomes. In the JIA group, the relative binding frequency to the marked proteins of the ciliary body was significantly higher than that to the marked proteins of the iris (Kruskal-Wallis test with *post-hoc* analysis: *p* < 0.05). In the healthy control and the JIAU group, the relative binding frequency to proteins of the different proteomes was similar (data not shown). In a next step, we conducted cluster analyses to get further insight into differences in the binding patterns and repertoires of serum antibodies to ocular tissue proteomes between the patient groups.

### Cluster Analysis

**Figure 3:**
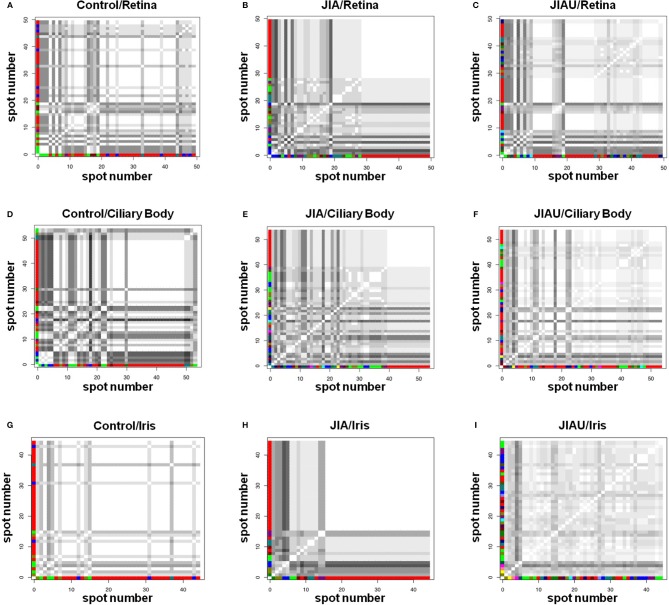
Cluster analysis represents the pairwise comparison of the protein spots labeled on the proteomes with respect to the binding pattern of patient sera. Two spots linked by a white square signifies 100% congruency. The darker a square, the greater is the heterogeneity between the two spots. The color code of the axes assigns spots of the same cluster. Coincidence matrixes **(A–I)** are described by the number of clusters, representing a measure of the variance with which the protein spots are bound by the different serum samples. **(A)** Healthy control/retina: 7 clusters required to cover 91.6% of the variance. **(B)** JIA/retina: 10 clusters required to cover 90.1% of the variance. **(C)** JIAU/retina: 13 clusters required to cover 90.9% of the variance. **(D)** Healthy control/ciliary body: 8 clusters required to cover 91.7% of the variance. **(E)** JIA/ciliary body: 15 clusters required to cover 91.5% of the variance. **(F)** JIAU/ciliary body: 15 clusters required to cover 91.5% of the variance. **(G)** Healthy control/iris: 5 clusters required to cover 94.8% of the variance. **(H)** JIA/iris: 8 clusters required to cover 90.0% of the variance. **(I)** JIAU/iris: 17 clusters required to cover 90.6% of the variance.

Cluster analysis comprises the pairwise comparison of the binding pattern of all spots marked on the respective proteome with each other. If two spots are bound exactly by the same sera, they are defined as maximally similar (white squares in Figure 3); if the spots are bound completely heterogeneously, they are defined as maximally dissimilar (black squares in Figure 3). Based on this similarity (coincidence matrix of binding patterns), the spots are grouped into clusters, using a technique of unsupervised pattern recognition. We determined the minimal number of clusters so that the between-class variance covered 90% of the total variance.

In all three proteomes (retina, ciliary body, and iris), the healthy control group showed the lowest number of clusters. In the retina and ciliary body proteome, only slight differences were observed between the JIA and JIAU group with respect to the number of clusters. However, in the iris, the JIAU group showed a higher number of clusters than the JIA and healthy control group (Figure 3). Therefore, cluster analysis indicated that serum antibodies targeted a widespread range of antigenic proteins isolated from ocular tissues in both JIA and JIAU patients, which was most pronounced in JIAU patients, particularly, with respect to iris tissue. This suggests a heterogeneous antibody repertoire to antigenic targets of the iris, particularly in JIAU patients.

### Single-Spot Analysis

**Figure 4:**
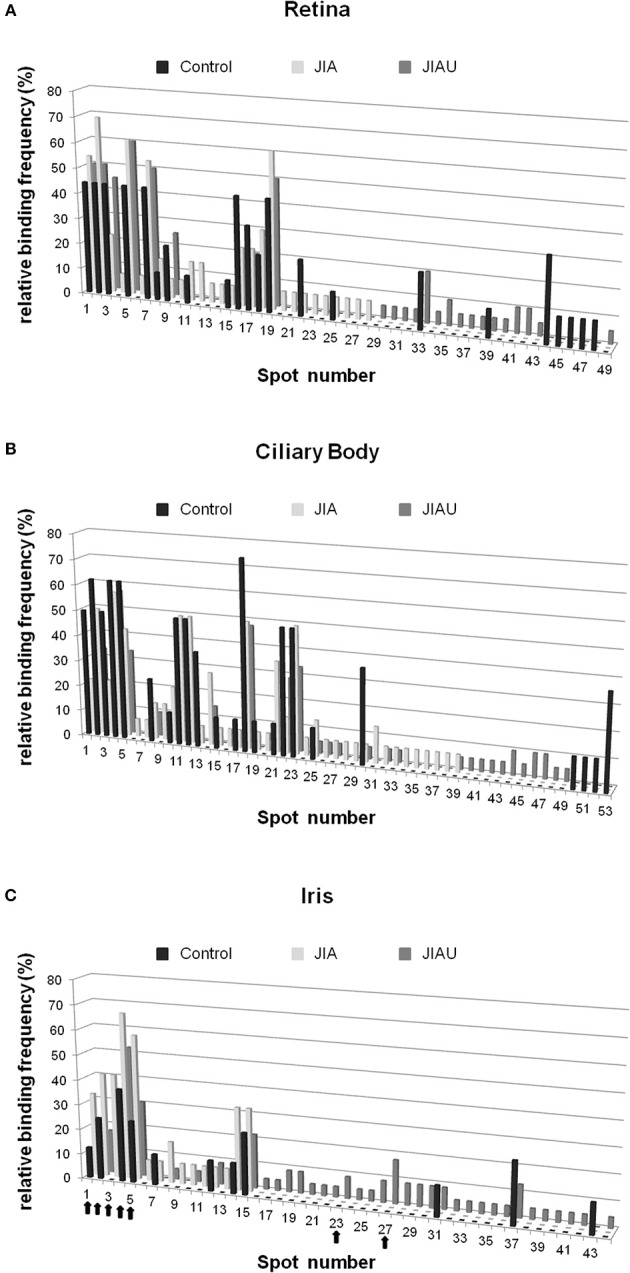
Single-spot analysis. For each protein spot of the **(A)** retina, **(B)** ciliary body, and **(C)** iris, the proportion of reactive sera of the patient groups and controls (relative binding frequency) was determined. The spot number corresponds to the spot labeling in Figure 1. Arrows highlight the protein spots that were selected for MS.

In a next step, we aimed to identify the proteins in spots that were bound most frequently by serum antibodies. In all groups, spots 1, 2, 3, 5, 7, and 19 of the retina proteome (Figure 1A) were frequently bound by serum antibodies. Each of them was bound by 44% of healthy control patient sera. The proportion of sera binding to these spots ranged from 45 to 60% in the JIAU group and from 23 to 69% in the JIA group (Figure 4A).

From the ciliary body proteome, spots 2, 4, and 18 (Figure 1B) were frequently bound in all patient groups. A proportion ranging from 63 to 75% of sera binding to these spots were found in the control group, 50 to 57% in the JIA group, and 33 to 57% in the JIAU group. Spot 5 was frequently bound by both healthy control sera (63%) and JIAU patient sera (33%), while spot 23 was frequently bound by both sera of the JIA (50%) and the JIAU (33%) group. Spots 11 and 12 were bound by 50% of JIA sera (Figure 4B).

Regarding the iris proteome (Figure 1C), spots 4 and 5 were found to be frequently bound in all patient groups. Spot 4 was bound by 38% of healthy control sera, 67% of JIA, and 52% of JIAU patient sera. Spot 5 was bound by 25% of healthy control sera, 58% of JIA, and 30% of JIAU patient sera. Further, spots 1 and 2 were bound by a considerable proportion of healthy control sera (13 and 25%, respectively) and JIA (33 and 42%, respectively) and JIAU patient sera (9 and 17%, respectively). Spot 3 was bound by 42% of the JIA patient sera and by 17% of the JIAU patient sera. Iris protein spots 23 and 27 were exclusively bound by JIAU patient sera (9 and 17%) (Figure 4C).

These iris protein spots, which were frequently bound by serum antibodies in each patient group (spots 1, 2, 3, 4, 5) or which were exclusively bound by JIAU patient sera (spots 23, 27), were subjected to MS analysis.

### Protein Identification

In JIA-associated uveitis, the intraocular inflammation site predominantly comprises the iris and ciliary body tissue (3, 4, 8, 14, 15). In the present study, we detected patient serum antibodies that target a broad range of proteins, particularly in the iris proteome, which was most pronounced in the JIAU group. To identify potential antigenic targets of the serum antibodies, we selected the most frequently bound iris protein spots on 2D gels in the three patient groups as well as spots exclusively bound by JIAU patient sera for MS analysis. MS results are summarized in Table 2. Protein spots frequently bound by patient sera of all groups contained tubulin beta chain (spot 1, Figure 1C), vimentin (spot 2, Figure 1C), ATP synthase subunit beta (spot 3, Figure 1C), actin (spot 4, Figure 1C), and L-lactate dehydrogenase B chain (spot 5, Figure 1C). Spots 23 and 27, which were exclusively bound by JIAU patient sera, were identified as heat shock cognate 71 kDa protein and keratin, respectively.

**Table 2:** Proteins assigned by MS analysis.

**Spot no^a^**	**Protein name**	**UniProt accession number**
1	Tubulin beta chain	Q767L7
2	Vimentin	P02543
3	ATP synthase subunit beta	Q0QEM6
4	Actin^b^	A0A0B8RTA2
5	L-lactate dehydrogenase B chain	P00336
23	Heat shock cognate 71-kDa protein^b^	P11142
27	Keratin^b^	F1SGG6

*Isoforms were not differentiated*.

a *Spot number corresponds to the spot labeling in Figure 1C*.

b *No porcine database entry available*.

## Discussion

With respect to the high prevalence of the oligoarthritis subtype and ANA positivity and low frequency of RF positivity, the JIAU patients included in the study conform to the demographic and clinical characteristics typically described for this uveitis entity (3, 5, 8). The JIA patients without uveitis selected for the study matched these characteristics, resulting in homogeneous study groups. Chronic anterior uveitis is the most common course of JIAU (8), which was also registered in the JIAU patients included in the present study. The frequent use of systemic and local anti-inflammatory treatment is in accordance with the recommendations for JIAU management (11, 20–22). In both JIA and JIAU patients a predominance of female sex was reported (3, 6). Correspondingly, the patient cohort of the present study, including the healthy controls, comprised a higher proportion of female patients. In the present study, the mean age differed significantly between the healthy control and the patient groups. Furthermore, at the time of sampling, JIA disease duration was significantly higher in JIAU patients than in JIA patients. However, statistically, no evidence was found that the patient age or JIA disease duration of the patients influences the binding pattern or binding frequency of serum antibodies to the proteome isolated from iris, ciliary body, or retina tissue (Supplement 1). In the present study, sampling from initially treatment-naïve patients of the different study groups or patients with the same basic therapy was not feasible, but would be desirable for future studies to exclude also any potential influence of anti-inflammatory therapies on autoantibody repertoires.

Due to the availability of porcine tissues and the high homology of porcine and human genes and proteins (23–25), we tested the binding of patient serum antibodies to the proteome isolated from porcine ocular tissues. MS data were analyzed with both the human and the porcine Uniprot database and showed similar results (data not shown). Thus, positive signals in western blot analyses reflected the binding of serum autoantibodies to autoantigens isolated from ocular tissues.

The data presented here demonstrate the presence of autoantibodies against ocular tissue proteomes in all patient groups, including healthy controls, which has been observed previously. Several other studies also report a complex repertoire of autoantibodies in normal human serum that target diverse antigenic structures (26–30). A functional role of autoantibodies has been suggested in physiological maintenance, homeostasis, and immune regulation, on the one hand, and pathological destructive autoimmune conditions, on the other (26, 31).

We analyzed the binding pattern of serum autoantibodies to ocular tissue proteomes by detecting autoantigenic proteins on 2D-gel spots and determining the patient sera reactivity to each of these spots. This procedure revealed an increased binding frequency of JIAU sera to iris protein spots. Furthermore, cluster analysis confirmed a high diversity of autoantibody specificities against the iris proteome, particularly in JIAU patients, implying that a broad range of autoantibodies against autoantigenic targets is present in the iris in JIAU. This is in line with our previous study that showed positive immunohistochemical staining after incubating ocular tissue sections with JIAU patient serum predominantly in the iris and ciliary body. Although the binding of serum autoantibodies to iris and ciliary body was pronounced in the JIAU group, it was not restricted to this uveitis entity, but was also common in patients with idiopathic anterior uveitis and, to a lesser extent, in JIA patients without uveitis (32).

Previous studies have shown that the iris is a major site of inflammation in anterior uveitis, which is the most common form of JIAU (8, 13, 14). Inflammation is associated with tissue damage, various forms of cell death, a high turnover of cells, and diverse modifications of molecules, leading to the formation and release of neo-autoantigens. Consequently, these circumstances may promote the diversification of epitope specificity of the immune response, including the repertoire of antibody specificities, called epitope spreading (33–36). In rheumatoid arthritis (RA), autoantibodies and epitope spreading of antibodies to citrullinated protein antigens are considered to play a critical role in the progression from preclinical to clinical disease states (35, 37, 38). Anti-citrullinated protein/peptide antibodies (ACPA) were also frequently shown in JIA patients. Indeed, children who were ACPA-positive and RF-negative were characterized by early age at disease onset (39). However, mass spectrometry analysis of the present study did not differentiate between isoforms or post-translational modifications of proteins such as citrullination or carbamylation.

Nevertheless, whether autoantibodies are causative for autoimmune inflammation or a consequence thereof, they may enhance and perpetuate inflammatory reactions by activating immune cells, endothelial cells, and fibroblasts in a proinflammatory and oxidative stress-related manner as shown for anti-endothelial cell antibodies (40–46). In experimental autoimmune uveoretinitis (EAU), immune serum from mice actively immunized with retinal antigens was shown to be unable to induce the uveitis model, but did increase disease severity after EAU induction by adoptive transfer of uveitogenic T cells and, therefore, modify the disease course (47). In contrast, collagen-induced arthritis (CIA) could be passively transferred to healthy recipient animals by injecting immunoglobulin concentrates of immune serum or anti-collagen antibodies systemically (48, 49). A crucial role of autoantibodies in JIAU is supported by studies that demonstrate B and plasma cells to be dominant cell types in the ocular inflammatory infiltrate, a high prevalence of ANA, and rituximab to be effective in the treatment of JIAU patients (3, 8, 14, 16, 50, 51). Furthermore, it was reported that anti-H3 histone serum antibodies and serum antibodies against different ocular antigens such as low-molecular-weight iris antigen or retinal S antigen correlated with uveitis occurrence (52, 53). In previous work of Deeg et al., an experimental procedure similar to that in our study was conducted to analyze the IgG antibody-binding profile to retinal proteome and to identify the proteins of spots that were selectively bound by equine recurrent uveitis serum autoantibodies. The retina- and pineal gland-specific protein cellular retinaldehyde-binding protein (cRALBP) was identified as a novel autoantigen, which is expressed in retinal Mueller glial cells and the RPE and was shown to be uveitogenic in horses and rats (18).

The protein spots that were exclusively bound by JIAU patient serum autoantibodies in the present study were identified as keratin and heat shock cognate 71-kDa protein (Hsc71). Despite the high proportion of ANA-positive patients in the JIAU cohort, no nuclear proteins were found to be among the identified autoantibody targets. In contrast to the autoantigen identified in the work of Deeg et al. (18), the expression of keratin and Hsc71 is widely distributed and not restricted to ocular tissues. However, ubiquitous proteins may represent autoantigenic targets in tissue-specific autoimmune reactions. Broekhuyse et al. demonstrated the induction of experimental autoimmune anterior uveitis (EAAU) by immunizing rats with bovine melanin antigen preparations from the skin, while the inflammation affected primarily the iris and ciliary body (54). A further study signified the ubiquitous protein type I collagen α-2 chain to be the uveitogenic constituent in melanin-associated antigen (55).

Hsc71 is a member of the heat shock protein 70 family (56). Heat shock proteins were implicated in various disorders and reported to be targets of autoantibodies in several diseases of a presumed immune-mediated or autoimmune nature (57–61). Correspondingly, serum antibodies against inducible 70 kDa heat shock protein were detected in different uveitis entities (62). Peptides derived from human heat shock proteins, which are recognized by T lymphocytes from Behcet's disease patients, were shown to be uveitogenic in rats and to induce anterior uveitis, which is a major manifestation of Behcet's disease (63). Serum autoantibodies against 65/70 kDa heat shock protein were also significantly increased in Behcet's disease (64). In a study of Kimura et al., autoantibodies against Hsc71 were detected in cerebrospinal fluid from patients with various neurologic diseases. However, the function and the pathogenetic relevance of the autoantibodies against the identified antigens were not known. A further concordance with our study was the detection of autoantibodies against several cytoskeletal proteins, such as vimentin or tubulin beta chain, that are abundant in intracellular compartments and that were neither specific to neural or ocular tissues nor to a patient group (19). In order to get more insight into the functional and pathogenetic relevance of the autoantibodies against the antigens identified in the present study and their potential as JIAU biomarkers, quantifying the respective autoantibodies in serum samples of the different patient groups or ocular fluids could be a promising approach. In this context, investigating disease specific changes in profiles and quantities of autoantibodies to a complex set of antigenic targets (e.g., by multi-analyte arrays) could be of great interest. Furthermore, our systematic procedure for identifying potential JIAU-specific autoantigens could be extended to protein spots bound by other antibody isotypes such as IgM in future studies.

Taken together, in the present study, we detected a broad range of autoantibodies that are directed against iris antigens, notably in JIAU patients. In JIAU, the iris is the primary site of inflammation, supporting a crucial role of autoantibodies and B cells in the pathogenesis of this uveitis entity. The extent to which the autoantibodies against the antigens that were identified in the present study might play a functional role—e.g., determining the disease course and severity–and serve as markers for JIAU or predictors for the risk of developing uveitis in JIA requires further investigation.

## Data Availability

The datasets for this study will be made available on request.

## Ethics Statement

The study design complies with the standards put forth by the Declaration of Helsinki. The study was approved by the local ethics committee of the Medical Association Westfalen-Lippe and the Westphalian Wilhelm University of Münster. All subjects provided written informed consent for peripheral blood collection.

## Author Contributions

MB, KWe, KWa, DB, CD, RD, SK, ST, MK, and AH designed the study and wrote the manuscript. MB, KWe, KR, CD, RD, DA, SK, and MK performed the experiments and analyzed the data. KWa and AH collected the peripheral blood samples and provided clinical data.

### Conflict of Interest Statement

The authors declare that the research was conducted in the absence of any commercial or financial relationships that could be construed as a potential conflict of interest.

## Abbreviations

JIA: Juvenile Idiopathic Arthritis
JIAU: Juvenile Idiopathic Arthritis-Associated Uveitis.
