# Supplementary material for: Identification of Ocular Autoantigens Associated With Juvenile Idiopathic Arthritis-Associated Uveitis
Source: Front Immunol. 2019 Aug 6;10:1793. doi: 10.3389/fimmu.2019.01793 (PMC6691058; doi:10.3389/fimmu.2019.01793)
Supplement: Supplementary file 1 [file Data_Sheet_1.PDF]

Supplement 1:
In order to statistically analyze a potential impact of the patients' age or the disease duration (JIA/uveitis) at the time of sampling on the binding of serum antibodies to the protein spots of the different proteomes (retina, ciliary body, iris), we divided the patients of the different groups (Control, JIA, JIAU, all [patient groups pooled]) into two subgroups according to their binding behavior. In one subgroup, a specific protein spot was bound by serum antibodies of the patients (subgroup „binding“). In the other subgroup, serum antibodies did not bind to the respective protein spot (subgroup „no binding“). We then compared the age/disease duration between these two subgroups (binding/no binding) by using a Mann-Whitney-U-test. This procedure was conducted for each protein spot of each proteome (retina: n=49; ciliary body: n=53; iris: n=44) and for each patient group (Control, JIA, JIAU, all).
Age at the time of sampling: a total of 382 tests was valid (i.e., sufficient cases in both subgroups (binding/no binding). No significant difference in the patients' age between the subgroups „binding“ and „no binding“ ($P>0.05$) in 99% of the tests.
JIA disease duration: a total of 97 valid tests. No significant difference in the JIA disease duration between the subgroups „binding“ and „no binding“ ($P>0.05$) in 92% of the tests.
Uveitis duration: a total of 64 valid tests. No significant difference in the uveitis duration between the subgroups „binding“ and „no binding“ ($P>0.05$) in 97% of the tests.
Conclusion: the binding of serum antibodies to a protein spot does not depend on the age or the disease duration (JIA/uveitis) of a patient.

Additionally, to analyze a potential impact of the patients' age or the disease duration (JIA/uveitis) at the time of sampling on the binding of serum antibodies to the protein spots, we substantiated the preceding statistical analyses by conducting logistic regression analyses calculating the odds ratio (OR) with 95% confidence interval (CI) for each patient group (Control, JIA, JIAU, all) and each protein spot of the different proteomes (retina, ciliary body, iris).
In all logistic regressions (Age at sampling, n=236; JIA disease duration, n=267; uveitis duration, n=178), OR=1 was within the CI.
Thus, a significant impact of the patient's age at sampling or the disease duration (JIA/uveitis) on the binding of serum antibodies to the protein spots can be excluded.
